# Understanding age and sex differentials in cancer incidence and mortality: An international population‐based study

**DOI:** 10.1002/ijc.70244

**Published:** 2025-11-10

**Authors:** Nolwen Rodet, Hana Zahed, Murielle Colombet, Freddie Bray, Valerie McCormack

**Affiliations:** ^1^ International Agency for Research on Cancer Lyon France

**Keywords:** cancer burden, global burden, sex differentials

## Abstract

Most epidemiologic analyses of male‐to‐female (M:F) ratios for cancer incidence have done so to gain aetiological understanding, focusing on age‐standardized M:F rate ratios for specific cancer sites. None have quantified the extent and timing, with respect to age, of sex differentials in the total cancer burden (all sites excluding non‐melanoma skin cancer). In the present study, using data from IARC's Cancer Incidence in Five Continents for 2013–17 (*N* = 60 countries) and GLOBOCAN mortality 2022 (*N* = 69), we estimated ages when the sex ratios peaked and reversed from a male to a female excess, or vice versa. Across all countries included, a common 4‐period pattern was observed. For incidence, period 1 featured an early‐life male excess up to age 21 years (region‐specific means ranged from 18– to 24), followed by a period 2 multi‐fold female excess lasting until age 59 (56–65) and peaking at a F:M of 2.4:1 (2.0–2.9) at age 41 (39–47), then period 3 with a large male excess peaking at M:F of 1.5:1 (1.2–1.6) at 73 (69–85) years. For the absolute burden alone (not rates) countries with long life expectancies experienced a 4th period of a female excess of cancers/cancer deaths at ≥85 years. These patterns were also present for mortality, but with a shorter period 2 duration. In summary, this study characterizes the four age periods of alternating sex differentials in the cancer burden, providing essential information to support sex‐appropriate allocation of cancer prevention and oncology resources.

## What's New?

Cancer incidence and mortality differ between males and females. Whether these differences fluctuate across life stages, however, remains largely unknown. Drawing on worldwide cancer registry data and mortality estimates, this study explored age‐related differences in cancer occurrence between the sexes. Globally, cancer patterns show four phases: a slight male excess in childhood, a female excess in early to mid‐adulthood, a male excess in later life, and—where female longevity is high—a return to female excess at very old ages. Marked excesses in early cancer risk in females extend survivorship and the period of vulnerability to late treatment effects.

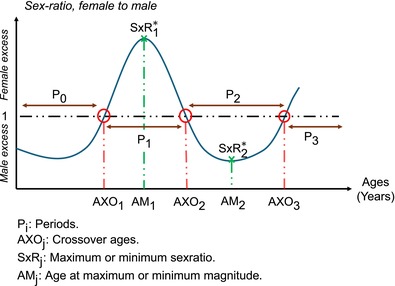

**Abbreviations**
AIC: akaike information criterion—a measure used in model evaluation
ASR: age‐standardized rate—a rate adjusted to a standard age distribution
CI5: cancer incidence in five continents—a series of standardized cancer registries
CI5‐XII: The 12th volume of *Cancer Incidence in Five Continents* series
F:M: female‐to‐male ratio—the proportion of females to males
GLOBOCAN: Global Cancer Observatory—a comprehensive database of global cancer statistics
HDI: Human Development Index—a composite statistic of life expectancy, education, and per capita income
IARC: International Agency for Research on Cancer
ICD‐10: International Statistical Classification of Diseases 10th Revision
M:F: male‐to‐female ratio—the proportion of males to females
NMSC: non‐melanoma skin cancer—refers to skin cancers excluding melanoma
ROI: region of interest—geographic or analytic area under study
SD: standard deviation—a measure of statistical variability around the mean
US: United States
WPP: world population prospects—United Nations demographic estimates
YLD: years lived with disability—years lived in ill health
YLL: years of life lost—years lost due to premature mortality

## 1 INTRODUCTION

Cancer is a leading cause of morbidity and mortality worldwide,^1^ with almost 20 million people diagnosed with cancer in 2022, and a corresponding 9.7 million people dying from the disease.^2^ Whether assessed as the absolute number of incident cases or deaths across all ages, or as age‐adjusted incidence or mortality rates, all‐cancer male to female (M:F) ratios are greater than unity, implying males are at excess risk of cancer and cancer death relative to females. However, this male excess does not hold across all ages; rather it has been noted that two‐thirds of cancers diagnosed worldwide at the ages of 20–49 years occur in females.^3^, ^4^ Despite this latter observation, there are very few systematic analyses examining sex differentials in cancer incidence and/or mortality according to age, as most aetiological studies focus on site‐specific M:F ratios across broad age groups or standardized for age.^5^, ^6^ One analysis of all‐cancer incidence rates in the United States in the period 1975–2004^7^ did report elevated incidence rates among females compared to males (M:F <1 or F:M>1) at ages 20–49, reversing to a tendency for higher rates in males than females at all other ages (M:F >1). A more comprehensive evaluation of these sex ratios across all ages and countries is needed to assess whether the US pattern holds elsewhere, so that sex‐specific allocation of the finite resources for cancer control to primary prevention, early detection and oncology services can be appropriately distributed.

To fill this gap in knowledge, the present study examines recorded incidence data from high‐quality cancer registries worldwide and national estimates of cancer mortality to systematically examine the ages at which males and conversely females experience excesses in cancer incidence and cancer deaths, both in terms of absolute numbers and rates. We estimate the ages at which the peak M:F and F:M ratios occur and how these ratios vary by world region and national levels of the Human Development Index (HDI^8^). We also examine whether the age‐related patterns of sex ratios remain after restricting the analysis to cancers common to both sexes.

## 2 METHODS

### 2.1 Data sources

We extracted incidence and mortality counts and rates by age and sex from high quality data sources, to estimate age‐specific sex ratios. For cancer incidence, we restricted analyses to high quality population‐based cancer registries compiled in Volume XII of *Cancer Incidence in Five Continents* (CI5‐XII), which refers to diagnoses mainly for the quinquennial period 2013–2017.^9^ It provides the number of cases and corresponding population sizes by cancer site, five‐year age group (up to 18 categories: 0–4, 5–9, …, 85+ years), and sex. Of the 460 registries in 65 countries included in CI5‐XII, we restricted analysis to 388 registries in 60 countries to ensure sex ratios by age were robust, aggregating registries by country (the unit of analysis), and excluding countries with small numbers defined as having recorded less than an annual average of 200 cancer cases in either sex (less than 1000 cases in 5 years in either sex). Five countries were excluded on this basis—Benin, Liechtenstein, Qatar, Seychelles, and, with a single registry included in CI5‐XII (Eastern Cape), South Africa—leaving 60 countries included, as listed in Supplementary Table S1.

For data on cancer mortality, we used IARC's GLOBOCAN 2022 database,^2^, ^10^ restricting to the 90 (of 185) countries whose estimates were of high quality, being derived from national rates projected to 2022. Thereafter, we excluded 7 countries with no cases in any sex‐5‐year age group and 14 countries with less than 1000 cancer deaths over 5 years for either sex: Cape Verde, Fiji, Guyana, Iceland, Bahamas, Luxembourg, Malta, Bahrain, Mauritius, Barbados, Saint Lucia, Suriname, Belize, and Brunei Darussalam (included countries are listed in Supplementary Table S2). We also extracted GLOBOCAN 2022 incidence estimates for the same 69 countries for direct comparisons of sex ratios between incidence and mortality. In both CI5 and GLOBOCAN, counts of cancer incidence/deaths were extracted for (i) all cancer sites except NMSC (International Classification of Diseases 10th revision [ICD‐10]: C00‐97 but C44), and were partitioned into one of three groups: (ii) cancers common to both sexes (excluding breast), (iii) female‐specific cancers (including breast), and (iv) male‐specific cancers. Female‐specific cancers comprised breast (as 99% occur in females), vulva, vagina, cervix uteri, corpus uteri, uterus unspecified, ovary, other female genital organs and placenta (ICD‐10: C50‐C58). Male‐specific cancers comprised penis, prostate, testis and other male genital organs (ICD‐10: C60‐C63). Cancers common to both sexes were all other sites (ICD‐10: C00‐C49, and C64‐C95).

For the third data source, in order to achieve the highest possible precision in estimating age differentials, we extracted population estimates by sex, country, and single year of age (from 0 to 100 years) from the World Population Prospects (WPP) for the period 2015 to 2021.^11^ This database provides official United Nations population estimates and projections, prepared by the Population Division of the Department of Economic and Social Affairs of the United Nations Secretariat.

### 2.2 Statistical analysis

All analyses were conducted at the country level, and for four measures of the cancer burden: numbers (counts) of incident cancers, numbers of deaths, incidence rates and mortality rates. In a data preparatory step, after aggregating across constituent registries for a given country, we first excluded all cases with missing age at diagnosis, which constituted 5.3% of the recorded incident cases in CI5‐XII and similar proportions by sex, thus having a marginal effect on the primary analysis.

Among the registries included in CI5‐XII, some do not provide the standard 18 age categories, but instead report a reduced number of groups, with the oldest category often encompassing all individuals aged 70+, 75+, or 80+. To harmonize these registries with others that use more detailed age breakdowns, we redistributed the number of cases from the uppermost category into the missing age groups. This redistribution was done proportionally based on the age distribution of the corresponding country's population, as reported in the 2015 WPP.

**FIGURE 1:**
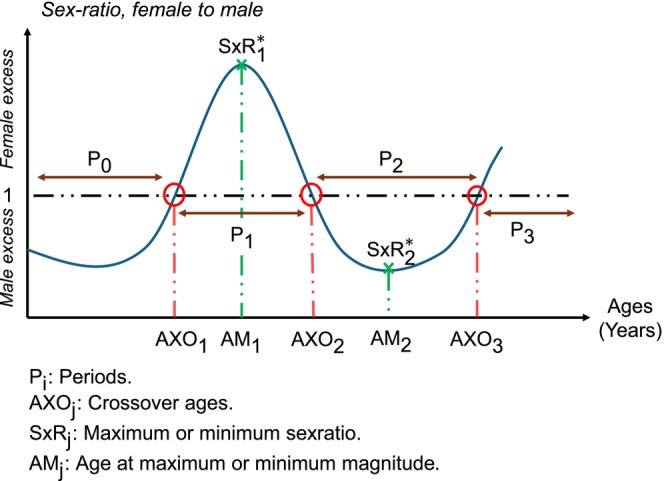
Schematic of the general pattern of incidence and mortality female to male (F:M) sex ratios with age and the present study's metric of interest at the reversal of and peak ratios.

Thereafter, the sex ratios for the four cancer burden measures were derived and visualized in 5‐year age categories to identify their major peaks and troughs. As shown in Figure 1, four distinct periods (P) of these sex ratios were generally observed: a childhood male excess (P_1_), followed by a pronounced (in relative terms) female excess in young to mid‐adulthood (P_2_), then a later‐life male excess beginning around the sixth or seventh decade of life (P_3_), and finally, in a subset of countries, a slight female excess re‐emerging after age 80 (P_4_).

To precisely estimate the ages at which these reversals and peaks occurred, we estimated counts and rates by single years of age, but only within the two or three consecutive 5‐year age groups where a reversal or peak was observed. These zones were defined as the regions of interest (ROI). Restricting the analysis to ROIs allowed us to achieve precise estimates without losing accuracy by modeling the entire age range. In this regard, Figure 1 presents the general form of the observed sex ratio patterns, illustrating the four periods (P_1_–P_4_). The crossover ages—points at which the sex ratio reverses at the end of each period—are denoted AXO*ⱼ* (with *j* ∈ {1, 2, 3}). The maximum magnitude of the F:M in period 2 and M:F ratio in period 3, along with the age at which the peak ratio occurs, are represented by ratio SxR_*k*_ occurring at age AM_*k*_, respectively (*k* ∈ {1, 2}).

To estimate sex‐ and country‐specific counts/rates by single year of age within each ROI, we fitted a Poisson regression model to the observed number of cases or deaths. The model included a cubic spline for age (using the median age of each 5‐year group as input) and the log of the population at risk as an offset. The number of knots was chosen to minimize the Akaike Information Criterion (AIC), which consistently indicated one knot across countries. Using this model, we predicted single‐year counts and rates for each sex and country, with the WPP single‐year population as the offset. Predictions were scaled so that the total number of cases/deaths within each 5‐year group matched the observed counts. These estimates were then used to calculate the F:M ratio by single year of age.

Crossover ages are then identified as those at which the F:M ratio shifts from below to above one (or vice versa). In the instance that the F:M fluctuated from below to above and below 1 (or vice versa) repeatedly within the ROI (Figures S1, 2, and S2), we fitted a linear regression of the log ratio on a cubic spline for age and predicted the age (AXO_*j*_) when the log ratio was zero (thus *F* = 1). Similar approaches were used to estimate the timing of the peak sex ratio, this time focusing on the age of the maximum and minimum ratios based on ratios calculated by single year of age.

**FIGURE 2:**
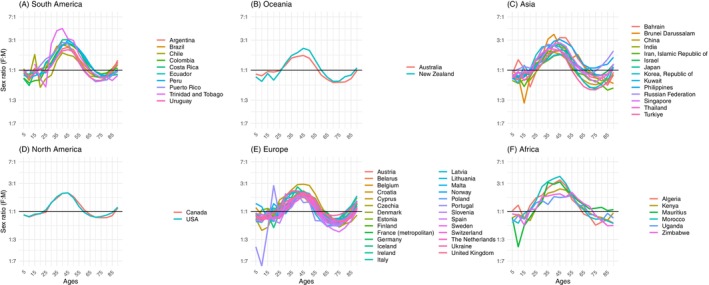
Sex–ratios (female: male) of absolute numbers of incident cancers (all sites excluding NMSC) versus age, based on country–specific raw data in 5–year age categories. Plots are divided by continent: (A) for South America, (B) for Oceania, (C) for Asia, (D) for North America, (E) for Europe, (F) for Africa. The *y*‐axis is on a log scale. Cancer Incidence in Five Continents Vol. X11, 2013–17.

## 3 RESULTS

### 3.1 Alternating pattern of sex differentials with age

**FIGURE 3:**
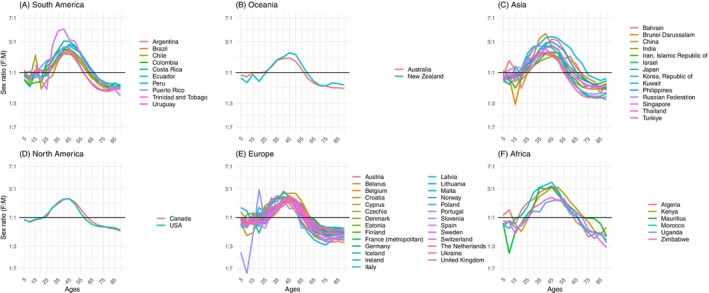
Sex‐ratios (female: male) of cancer incidence rates (all sites excluding NMSC) versus age, based on country‐specific raw data in 5‐year age categories. Plots are divided by continent: (A) for South America, (B) for Oceania, (C) for Asia, (D) for North America, (E) for Europe, (F) for Africa. The *y*‐axis is on a log scale. Cancer Incidence in Five Continents Vol. X11, 2013–17.

Of the 60 CI5 countries included, most registries were in Europe (27 countries), Asia (13) and a smaller number from South America (9) and Africa (6), whilst North America and Oceania have only a few large populous countries. In total, analyses included 14,577,450 incident cases in men, 13,258,622 in females (a global crude F:M ratio of 0.9). Figure 2 plots the sex ratios of the counts of new cases by 5‐year age group. The global pattern previously illustrated in Figure 1—characterized by four alternating periods of male and female excess—can be observed consistently across nearly all countries. This pattern is further reflected in Figure 3, which presents incidence rate ratios, showing similar oscillations in sex differentials as those seen in absolute incidence counts, with the exception of the absence of Period 4 (P_4_). Comparable trends were also evident in mortality metrics, both in terms of absolute numbers (Figures S1 and S3). Each period is described below.

### 3.2 Period 1: Childhood male excess

**FIGURE 4:**
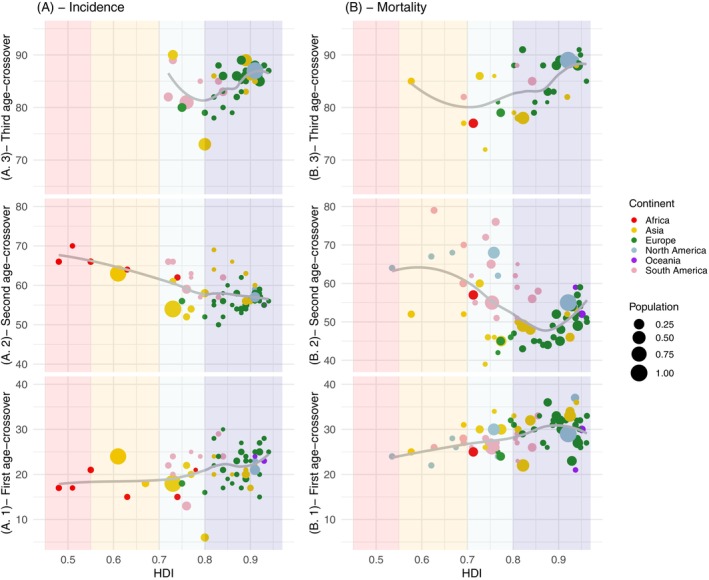
Crossover ages when F:M ratios reverse for: (A) number of cases and (B) number of deaths, according to each country's level of human development index. Crossover ages subplots are numerated as (A.1) and (B.1) for the first crossover age, (A.2) and (B.2) for the second crossover age, and (A.3) and (B.3) for the third crossover age.

Childhood cancers constitute the first period of interest (P_1_) where a male excess was seen, with an average M:F ratio for absolute incidence at ages 0–14 years of 1.2:1, and which was also present for incidence rates (Figure 3). As shown in Supp. Figure S1 the rarity of childhood cancer deaths led to large variations in the sex ratio, but as for incidence, this ratio pointed to a male excess in deaths across all continents. The duration of P_1_ lasts to a mean of 21.2 years (SD 4.2) for incident cancers (absolute numbers) and to 28.7 years (SD 3.6) for numbers of deaths (Table 1), however for both metrics, it terminated earlier in low than in high‐income countries (Figure 4). For example, the number of females diagnosed with cancer exceeded those for males from a mean age of 15 years in Algeria and Morocco, but not until age 28 years in Switzerland.

**TABLE 1:** Characteristics of the age profile of sex ratios in cancer incidence and mortality counts and rates, overall and by continent worldwide.

		Absolute numbers of cases							Incidence rates					
		Crossover Ages when F:M ratios reverses			Peak F:M and M:F ratios and their timing				Crossover Ages when F:M ratios reverses		Peak F:M and M:F ratios and their timing			
		First	Second	Third	Age		Magnitude		First	Second	Age		Magnitude	
		AXO1	AXO2	AXO3	AM1	AM2	SxR1 (F:M)	SxR2 (M:F)	AXO1	AXO2	AM1	AM2	SxR1 (F:M)	SxR2 (M:F)
Mean (SD)														
All countries (*N* = 60)		21.15 (4.2)	58.6 (4.6)	84.7 (3.6)	41 (5.1)	72.8 (6.7)	24:10	15:10	21.15 (4.2)	58.6 (4.6)	40.8 (4.5)	86.2 (12.8)	24:10	20:10
By continent (No. countries)														
Africa (6)		17.7 (2.7)	65.6 (3)	‐	47.3 (5.2)	85 (8.9)	29:10	17:10	18.3 (4.4)	64.8 (3.2)	43.5 (3.3)	92.3 (12.7)	30:10	29:10
Asia (14)		19.4 (4.4)	59.7 (5.2)	84.6 (5.6)	40.6 (5.3)	75.5 (5.8)	25:10	16:10	18.1 (4.9)	59.7 (6.1)	40.4 (4.2)	81.2 (8.8)	26:10	19:10
Europe (27)		22.2 (4.1)	55.9 (2.6)	84.6 (3.2)	39.5 (5)	69 (3.8)	21:10	16:10	21.3 (4.4)	55.4 (2.6)	40.1 (5.1)	85.1 (14)	21:10	20:10
North America (2)		22 (1.4)	58 (1.4)	87.5 (0.7)	43.5 (0.7)	69 (4.2)	21:10	13:10	20.5 (2.1)	57.5 (2.1)	44 (0)	100 (0)	21:10	18:10
Oceania (2)		23.5 (0.7)	56.5 (2.1)	88	42 (2.8)	69.5 (2.1)	20:10	15:10	22.5 (0.7)	55 (1.4)	42 (2.8)	87 (18.4)	19:10	17:10
South America (9)		22.3 (4.4)	62 (3.7)	84.2 (2.9)	41 (3.2)	75 (3.7)	28:10	13:10	22.2 (4.8)	60.4 (3.2)	40.4 (3.8)	89.8 (12.7)	27:10	20:10

		Absolute numbers of deaths							Mortality rates					
		Crossover Ages when F:M ratios reverses			Peak F:M and M:F ratios and their timing				Crossover Ages when F:M ratios reverses		Peak F:M and M:F ratios and their timing			
		First	Second	Third	Age		Magnitude		First	Second	Age		Magnitude	
		AXO1	AXO2	AXO3	AM1	AM2	SxR1 (F:M)	SxR2 (M:F)	AXO1	AXO2	AM1	AM2	SxR1 (F:M)	SxR2 (M:F)
Mean (SD)														
All countries (*N* = 69)		28.7 (3.6)	53.8 (8.6)	84.6 (4.8)	37.1 (4.4)	69.8 (8.5)	20:10	15:10	28.4 (3.7)	53 (7.3)	37 (4.2)	75.7 (11.4)	20:10	18:10
By continent (No. countries)														
Africa (1)		25	57	77	30	69	27:10	12:10	25	55	30	74	28:10	18:10
Asia (15)		30.1 (3.8)	49.3 (5)	82.4 (5.6)	37.2 (5.6)	67.7 (7.3)	18:10	17:10	29.5 (4.1)	48.9 (5.4)	36.6 (5.5)	72.2 (9.8)	18:10	21:10
Europe (27)		29.7 (3.2)	48.6 (4.6)	85.8 (4.1)	35.9 (2.8)	64.4 (6.6)	17:10	15:10	29.2 (3.3)	49 (4.8)	35.8 (2.5)	68.9 (6.2)	18:10	17:10
North America (8)		27.5 (4.7)	62.6 (6)	89 (0)	38.6 (5.8)	79.6 (6.2)	21:10	15:10	27.5 (4.9)	61.1 (4.4)	37.9 (5.7)	88.3 (13.3)	20:10	19:10
Oceania (2)		25.5 (6.4)	55.5 (5)	‐	39.5 (6.4)	78.5 (2.1)	19:10	14:10	25.5 (6.4)	54 (2.8)	39.5 (6.4)	81.5 (3.5)	18:10	16:10
South America (16)		27 (2.1)	62.3 (8.4)	85 (3)	38.7 (3.8)	75.4 (6.4)	27:10	14:10	26.9 (2.2)	59.7 (6.4)	38.7 (3.8)	83.6 (10.2)	27:10	18:10

*Note*: The characteristics include the ages of peaks and reversals in the M:F ratios across the four periods: Childhood male slight excess, young–mid adult excessive female excess, old large male excess and for absolute numbers, an elderly female excess. F:M: number of cases/deaths in women out of the number of cases/deaths in men. M:F: number of cases/deaths in men out of the number of cases/deaths in men. Cancer cases are taken from CI5 volume XII, and cancer deaths from GLOBOCAN 2022. Cancer cases were diagnosed between 2013 and 2017.

### 3.3 Period 2: Young‐middle‐aged adult life female excess

Young and middle adult life featured a female excess of cancer regardless of the metric utilized. For incident numbers, across all countries combined, period P_2_ commenced at age 21 and peaked at 41 years with a F:M of 2.4:1 for new cases and at a smaller peak ratio at a slightly younger age (37) for deaths. On a relative scale, this peak F:M ratio was highest in Africa, at 2.9:1, but even in regions where the relative female excess was lowest (Europe and North America) at its peak at age 40–44 years there were two females diagnosed with cancer for every diagnosis in men, and 1.6 cancer deaths in females (1.2 in N. America) for each one in males (Table 1). In total, this P_2_ female excess period lasted 37.4 years (all countries) for incident cases and 25.1 years for cancer deaths, that is, lasting until the mid to late 50s and early 60s (Figure *4*). In relative terms and for both incidence and mortality, the young to middle age female excess period was more pronounced, started earlier and finished later in lower‐income countries.

### 3.4 Period 3: Later adult life male excess

The late male excess period (P_3_) commenced at a mean age of 58.6 years (SD 4.6) (Table *1*) for the number of incident cases and earlier, from age 53.8 (SD 8.6) years, for deaths. On this relative scale, the mean F:M ratio at peak occurred at a mean of 72.9 years (SD 6.7) at which point there were 1.5 incident cases in males for every 1 in females, whilst for mortality the peak M:F ratio was at 70 years (8.5), with 1.5 deaths in males for 1 in females. Note, however that although all countries experienced this period P_3_ when considering incidence rates, two countries did not see a male excess of the absolute number of incident cases beyond the childhood period, that is, the Philippines and Mauritius.

### 3.5 Period 4: Elderly female excess period in absolute incidence and deaths

When considering absolute cancer incidence and mortality (but not rates), at elderly ages owing to their longer life expectancy, there are more female than male cancer cases and deaths. This last period begins at a mean age of 85.0 years (SD 3.6) for incident cases and 84.6 (SD 4.8) for deaths (Table *1*). As shown in Figure 4, this last period can only be seen in higher‐income countries.

### 3.6 Further sex comparisons

The above summaries focus upon age‐specific sex ratios. Large ratios can represent small absolute excesses when rates/numbers are low and vice versa. Thus, in Supplementary Figure S4 we also plot the absolute excess incident cases/cancer deaths by age (relative to the total cancer burden [both sexes]), which reveals that, contrasting to the P_2_ female excess that was largest on the ratio scale, in Europe, Asian, North America and Oceania, the absolute period $P_{3}$ excesses were larger for men. Africa and South America were exceptions to this, especially Africa where the younger population pyramid means that the male excess at older ages does not translate to a large proportion of the total cancer burden.

**FIGURE 5:**
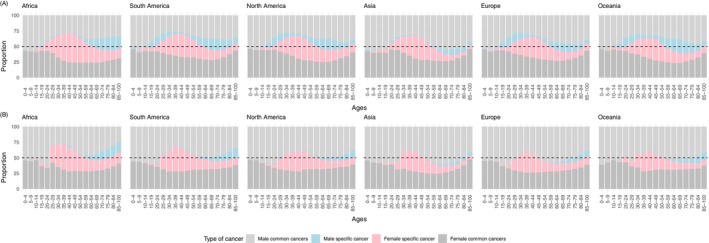
Proportions of the age‐specific cancer burden by common cancers in both sexes in men and women, as well as female and male‐specific cancers: (A) Absolute incidence; and (B) absolute number of deaths.

Decomposing the cancer burden into four groups (male‐specific cancers; sex‐common cancers in men; female‐specific cancers; sex‐common cancers in females) reveals the varying contributions to the four periods identified (Figures 5, S5). Notably, the childhood male excess (P_1_) is occurring in cancers that are common to both sexes. Then in the P_2_ period the large female excess coincides with the dominance of female‐specific cancers (notably breast and cervix). For incidence only, this period starts later in North America, Europe and Oceania than in other regions of the world due to the emergence of male‐specific cancers (notably testes) in the early twenties, counterbalancing the female‐specific cancers. Details on the starting ages and magnitudes for each period by country are available in Supp. Tables S3 and S4. In the P_3_ period, with the exception of Asia, a substantial proportion of the male excess comes from male‐specific cancers (notably prostate), which form a substantial (>15%) percentage of the above 60 cancer incidence burden (Figures 5, S5–S7).

## 4 DISCUSSION

This analysis provides, to our knowledge, the first international study examining the sex differentials of overall cancer occurrence by age, identifying the ages when the differentials are at their most *extreme* (in relative terms) and critically when they reverse in direction. In general, there were four successive periods, commencing with a childhood male excess, followed by a very large (in relative terms) female excess at young to middle ages. Subsequently, there is a large (in absolute terms) male excess until old age. Thereafter in countries with high life expectancy in females, there was a final female excess, in absolute numbers. This last reversal does not exist in rates, and the second male dominance remains. The duration of each period was dependent on a country's level of development, with the cancer burden disproportionately affecting females for longer periods, throughout their reproductive lives, in low‐ and middle‐income countries. In comparison to the previous US study by Cook et al,^7^ our results were very similar, with the P_2_ female excess period from 20 to 60 years. However, the magnitude of the peak sex ratio during this period was shallower than those found here. This may have been due to the wider calendar period (1975–2004) in their study.

Linked to sex differentials in the cancer burden by age is of course the years of life lost (YLL) and years lived with disability (YLD),^11^, ^12^, ^13^ thus the large female excess at younger and middle ages will be reflected in *a* large female excess of YLL and YLD. Indeed, in a 2012 analysis of all deaths in Norway, a consistent picture emerged. Brustogun et al. highlighted these opposing sex imbalances of the cancer burden, whereby the percentage of deaths due to cancer was lower in females than in males (23% vs. 29%); however in terms of YLL, the reverse occurred: 38% of YLL in females was due to cancer deaths, but only 33% in men.^12^ This disparity is not solely attributable to the longer life expectancy in females; rather as illustrated here, a greater proportion of female cancer deaths occur before the age of 55. Reporting YLL accentuates the effect of cancer on society. Indeed, YLL can capture the loss of productive years, which has direct implications for workforce participation, caregiving burdens, and healthcare costs.^14^, ^15^

In the present study, our main focus has been on the absolute number of new cancer cases and cancer deaths, since our primary perspective pertained to informing sex differentials in resource allocation for cancer, rather than aetiological insights. This absolute metric can provide an assessment that is masked by age‐standardization. For example, when examining age‐standardized incidence or mortality rates in Africa, we see that the general trend of a larger female excess in incidence rates (F:M based on GLOBOCAN ASRs of 1.12:1) than for mortality rates (1.00), which occurs because cancers diagnosed in females tend to be associated with more favorable prognoses than those diagnosed in men.^16^, ^17^ However, the ratio of 1.00 for the F:M mortality rate ratio standardizes Africa's young population to a much older one, artificially inflating weights of older ages when cancer mortality rates in males are high. This age‐standardized mortality rate ratio masks the fact that there are more cancer deaths in females than in males in Africa, considering all ages combined and acutely during young–mid adult life.

The impact of the present study lies in the possibility to channel cancer resources more efficiently and fairly by sex and age at a national scale. For the three to four decades during young/middle adulthood, females are disproportionately affected by cancer regardless of setting; thus a proportional weighting of secondary and tertiary prevention services toward females—for example, in early detection and health promotion, female wards, diagnostics specific to female cancers (mammographic and colposcopic exams)*—*would seem justified. The male cancer excess in later life is known to be driven by male‐specific cancers (prostate) as well as the large excess of cancers for which strong *risk* factors include those with sex‐patterned exposure gradients. These risk factors include tobacco, alcohol, and the many occupational carcinogens, often in combination.^4^, ^7^, ^18^, ^19^, ^20^ A male‐orientated allocation of resources for primary prevention of such exposures may be considered and given the latency period of carcinogenesis as well as the challenges to reverse life‐long habits, such efforts will be needed decades before the male excess commences. Moreover, as cancer trends evolve, notable shifts are expected. In some high‐income countries, lung cancer incidence among men is declining, while cervical cancer incidence among women is decreasing in low‐ and middle‐income countries due to vaccination programs. These changes will also affect the age patterns of cancer incidence, highlighting the need to monitor them closely. Such monitoring will be essential to adapt public health campaigns and policies to the evolving epidemiological and socio‐economic context, as HDI dynamics are also expected to change.

Finally, the appearance of an elderly female excess period (P_4_) is due to the sex‐imbalanced population structure at these ages. It would be interesting to understand when this last period first occurred historically, utilizing older long‐standing cancer registries, thus helping to anticipate the evolution of the sex‐specific cancer burden not only according to age, but also by transitioning socio‐economic background of a country. In contrast, there are perhaps fewer implications of a childhood male excess period, as it is relatively small and childhood cancer services are not generally segregated by sex.

As evidenced here, the marked excess of cancers among females at young to middle ages is a universal phenomenon affecting all countries. The implication of cancers at these relatively young ages is also immense as highlighted in the 2023 Lancet Commission on “Women, power and cancer.”^21^ The young female excess cancer risk also means that females with a lived experience of cancer have a longer survivorship period, when the late effects of cancer and its treatment can impact quality of life. Beyond the immediate impact on a female's life during the acute cancer therapeutic phase, there can also be negative impacts on her fertility, family and society.^22^, ^23^, ^24^, ^25^ For example, there are 7 million maternal orphans worldwide due to cancer.^26^ Failing to avert these deaths, universal health coverage could at least alleviate the cycle of poverty associated with them. Future attention to the specificities of the large and alternating sex differentials in the cancer burden is needed to tackle these cancer inequalities, whilst the present study provides the first necessary step to better quantifying these differentials.

## AUTHOR CONTRIBUTIONS

**Nolwen Rodet:** Methodology; visualization; writing – review and editing; software; writing – original draft; formal analysis. **Hana Zahed:** Methodology; writing – review and editing; formal analysis. **Murielle Colombet:** Writing – review and editing; data curation. **Freddie Bray:** Writing – review and editing; validation. **Valerie McCormack:** Methodology; writing – review and editing; supervision; validation.

## CONFLICT OF INTEREST STATEMENT

The authors declare no conflict of interest.

## Supporting information


**Data S1:** Supporting Information.

## Data Availability

All data used in this analysis are publicly available. Cancer Incidence in Five Continents, Vol. XII numbers are available from the International Agency for Research on Cancer's Cancer Incidence in Five Continents (CI5) Volume XII website: https://ci5.iarc.who.int. Accessed 04/08/2024. GLOBOCAN incidence and mortality estimates are available from the International Agency for Research on Cancer's Global Cancer Observatory: https://gco.iarc.fr/. Accessed 04/08/2024. The United Nations World Population Prospects 2015 and 2021 annual estimates of population by single age: https://population.un.org/wpp/downloads?folder=Standard%20Projections&group=Population. Accessed 04/08/2024. Further information is available from the corresponding author upon request.
