# Supplementary material for: Understanding age and sex differentials in cancer incidence and mortality: An international population‐based study
Source: Int J Cancer. 2025 Nov 10;158(8):2102–10. doi: 10.1002/ijc.70244 (PMC12922643; doi:10.1002/ijc.70244)
Supplement: Supplementary file 1 — Data S1: Supporting Information. [file IJC-158-2102-s001.pdf]

Understanding age and sex differentials in cancer incidence and mortality: an international population-based study.

Nolwen Rodet, Hana Zahed, Murielle Colombet, Freddie Bray, Valerie McCormack

SUPPLEMENTARY MATERIAL

List

Table S1 - Registries from CI5-XII used.

Table S2 - Countries included in the mortality analysis from GLOBOCAN 2022.

Figure S1: Female to male ratios of the number of deaths by age, based on higher quality data in Globocan 2022.

Figure S2 - Proportions of common sexes and sex-specific cancer deaths in global cancer deaths through age groups.

Figure S3: Sex-ratios (female:male) of absolute numbers of cancer deaths (all cancer sites excl. NMSC) versus age, based on country-specific raw data in 5-year age categories.

Figure S4: Difference of cases and deaths between females and males, scaled by total number of cases or deaths.

Figure S5– Proportions of the age-specific cancer burden by common cancers to both sexes in men and in women.

Figure S6: Sex-ratios (female:male) of absolute numbers of incident cancers (cancers sites common to both sexes) versus age, based on country-specific raw data in 5-year age categories.

Figure S7: Sex-ratios (female:male) of absolute numbers of deaths (cancers sites common to both sexes) versus age, based on country-specific raw data in 5-year age categories.

Table S3 - Summary table of age-crossovers, maximum sex-ratio and age at the maximum for each country, in incidence.

Table S4 -Summary table of age-crossovers, maximum sex-ratio and age at the maximum for each country, in mortality.

.

Table S1 - Registries from CI5-XII included in incidence analyses

Country	Name of the registry
**Africa**	
Algeria	Tizi Ouzou, Batna
Benin	Benin, Cotonou
Kenya	Nairobi, Eldoret
Mauritius	Mauritius
Morocco	Morocco, Casablanca
Seychelles	Seychelles
South Africa	Eastern Cape
Zimbabwe	Harare: African, Bulawayo: African
Uganda	Kyadondo County, Gulu
**Asia (except China which is at the end of the table)**	
India	*Tripura; Mizoram; Kollam; *Dindigul, Ambilikkai; *Bhopal; West Arunachal; *New Delhi; *Manipur; *Sangrur district; *Tamil Nadu; Barshi, Paranda, and Bhum; Mumbai; Chandigarh; *Chennai; Dibrugarh; SAS Nagar; Bangalore; Meghalaya; Poona; Wardha; Ahmedabad, Urban; Aurangabad; Kamrup Urban District; Trivandrum;
Iran (Islamic Rep. of)	Iran
Israel	Israel
Japan	Japan
Rep. of Korea	Rep. of Korea
Kuwait	Kuwait
Bahrain	Bahrain: Bahrainis
Philippines	Philippines, Manila
Qatar	Qatar: Qatari
Russian Federation	Arkhangelsk; Karelia; Pskov; *Samara; Kaliningrad; Murmansk; *Komi Republic; Vologda Region; *Orenburg;
Singapore	Singapore
Thailand	Songkhla; Bangkok; Lopburi Province; Lampang; *Chiang Mai; *Khon Kaen;
Türkiye	Bursa; Trabzon; Malatya; Antalya; *Erzurum; *Edirne; Samsun; Eski?ehir; Gaziantep; *Izmir
Brunei Darussalam	Brunei Darussalam
**Europe**	
Belarus	Belarus
Croatia	Croatia
Cyprus	Cyprus
Czechia	*Czech Republic
Denmark	Denmark
Estonia	Estonia
Finland	*Finland
France	*Gironde; *Calvados; *Bas-Rhin; *Loire-Atlantique; *Poitou-Charentes; *Vendée; *Territoire de Belfort; *Lille-Métropole; *Haut-Rhin; *Tarn; *Somme; *Limousin; *Hérault; *Isère; *Manche; *Doubs;
Germany	Bavaria; North Rhine-Westphalia; Rhineland-Palatinate; Hamburg; Schleswig-Holstein; Baden-Württemberg; Bremen; Saarland; Lower Saxony;
Iceland	Iceland
Ireland	Ireland

Italy	*Tuscany; South Tyrol; *Brescia; *Benevento; Basilicata; Puglia; Milan; *Lombardy, South, Pavia; *Naples South; Mantova and Cremona; *Nuoro; *Syracuse; *Salerno; *Sondrio; *Naples Centre; Marche; Emilia-Romagna; *Palermo; Trento; Calabria; *Aosta Valley; Brianza; Varese; *Caserta; Umbria; Veneto; *Catania, Messina and Enna; Turin; *Ragusa and Caltanissetta; Genova; *Naples North; Sassari; *Avellino; Friuli-Venezia Giulia; Bergamo; Molise;
Austria	Austria
Latvia	Latvia
Liechtenstein	*Liechtenstein
Lithuania	*Lithuania
Malta	Malta
Netherlands	*The Netherlands
Belgium	*Belgium
Norway	Norway
Poland	Poland, Kielce
Portugal	*Portugal, Azores
Slovenia	Slovenia
Spain	Tarragona; Navarra; La Rioja; Castellòn; Canary Islands; Granada; Salamanca; Asturias; Basque Country; Murcia; Girona
Sweden	*Sweden
Switzerland	Basel; *Neuchâtel and Jura; Vaud; Geneva; Ticino; *Graubünden Glarus; *Lucerne; Zurich and Zug; Valais; Aargau; *Fribourg; East; Berne Solothurn;
Ukraine	Ukraine
United Kingdom	England; Northern Ireland; Scotland; Wales
**North America**	
Canada	Saskatchewan; *Yukon; *New Brunswick; Alberta; *Newfoundland; *Prince Edward Island; Quebec; Ontario; British Columbia; *Manitoba
USA	USA, New Mexico; Connecticut; Iowa; NPCR
**Oceania**	
Australia	NSW/ACT; South Australia; Western Australia; Tasmania; Northern Territory; Queensland; Victoria
New Zealand	New Zealand
**South America**	
Chile	Region of Antofagasta , Valdivia
Colombia	Manizales; Bucaramanga; Pasto; Cali
Costa Rica	Costa Rica
Ecuador	Quito; Manabì; Guayaquil
Argentina	Entre Rìos Province; Mendoza
Peru	Peru, Lima
Puerto Rico	USA, Puerto Rico
Brazil	Recife; Aracaju; *Jaù; *Curitiba; Goiânia; João Pessoa; Belo Horizonte; *Barretos; *Campinas;
Trinidad and Tobago	Trinidad and Tobago
Uruguay	Uruguay
**Asia - China**	

China	*Hengfeng County, Yancheng , *Nantong City; Xianju County; Zhuanghe City; Ruyang County; Yunmeng County; Wu'an City; Ma'anshan City; Cixi City; Kunshan City; Sheyang County; Yanshi City; Shanghai City; Fuqing City; Luoding City; Yangquan City; Beijing City; Fangcheng County ; Longquan City; Xining City; Yueyanglou District, Yueyang Ci; Qidong County; Feixi County; Lucheng District, Wenzhou City; Xiping County; Qianxi County; Neixiang County; Faku County; Dancheng County; Liangshan County; Mengjin County; Ya'an City; Ci Xian County; Gaomi City; Luquan City; Rugao City ; Lianshui County; Anguo City; Yong'an City; Qingzhou City; Jiulongpo District, Chongqing; Zhuhai City; Yi'an District, Tongling City; Nanxiong City; Jinan City; Zhaoyuan City; Yakeshi City ; Nanhai District, Foshan City; Jiangyin City; Wuzhou City; Rural areas of Shanghai City; Yucheng County; Yinzhou District, Ningbo City; Wanzai County; Linqu County; Kaihua County; Zhongxiang City; Shangyu District, Shaoxing City; Hongshan District, Chifeng City; Guanyun County; Shunde District, Foshan City; Zhangjiagang City; Jintan District, Changzhou Cit; Zhongshan City; Donghai County; Liyang City; Xiangfu District, Kaifeng City; Jingxian; Linzhou City; Guanghan City; Jiange County; Liuzhou City; Macheng City; Shapingba District, Chongqing; Chongqing Yuzhong District; Jiayu County; Arun District; Yongkang City; Xishan District, Kunming City; Luoyang City; Yunyang District, Shiyan City; Song County; Zhengding County; Xuyi County; Qingyang District, Chengdu City; Ganyu District, Lianyungang Ci; Xin'an County; Jiangmen City; Luohe City; Wuhan City; Hai'an City; Linhe District, Bayannur City; Jingtai County; Ewenkizu Zizhiqi; Binhai County; Langzhong City; Huaiyin District, Huai'an City; Tengzhou City; Feicheng County; Suzhou City; Xinzhou District, Shangrao Cit; Duanzhou District, Zhaoqing Cit; Yanji City; Ganzhou District, Zhangye City; Xinji City; Hongta District, Yuxi City; Guangzhou City; Baoding City; Hailar District, Hulun Buir Cit; Xinluo District, Longyan City; Haimen City; Meihekou City; Cangzhou City; Wuhu City; Jiaxing City; Rudong County; Zanhuang County; Wangdu County; Hepu County; Dongguan City; Wuxi City; Gong'an County; Guang'an District, Guang'an Ci; Lianyungang City; Dongtai City; Anshan City; Hebi City; Yongding District, Longyan Cit; Donggang City; She Xian County; Shan County; Yiyuan County; Hangzhou City; Dawukou District, Shizuishan C; Dafeng District, Yancheng City; Changzhou City; Dalian City; Haining City; Hefei City; Gaocheng City; Hengdong; Danyang City; Jiyuan City; Anfu County; Guilin City; Honghu City; Yanting County; Changfeng County; Nangang District, Harbin City; Shijiazhuang City; Yangzhong City; Yingdong District, Fuyang City; Dehui City; Aohan District, Chifeng City; Dangtu County; Jiashan County;

Table S2 - Countries included in the mortality analysis from GLOBOCAN 2022

Continent	Country	Country code
Africa	South Africa	710
Asia	Armenia	51
Asia	Azerbaijan	31
Asia	Georgia	268
Asia	Iran, Islamic Republic of	364
Asia	Israel	376
Asia	Japan	392
Asia	Kazakhstan	398
Asia	Korea, Republic of	410
Asia	Kyrgyzstan	417
Asia	Mongolia	496
Asia	Russian Federation	643
Asia	Singapore	702
Asia	Syrian Arab Republic	760
Asia	Turkiye	792
Asia	Uzbekistan	860
Europe	Austria	40
Europe	Belgium	56
Europe	Bulgaria	100
Europe	Croatia	191
Europe	Czechia	203
Europe	Denmark	208
Europe	Finland	246
Europe	France (metropolitan)	250
Europe	Germany	276
Europe	Greece	300
Europe	Hungary	348
Europe	Ireland	372
Europe	Italy	380
Europe	Lithuania	440
Europe	Moldova	498
Europe	Norway	578
Europe	Poland	616
Europe	Portugal	620
Europe	Romania	642
Europe	Serbia	688
Europe	Slovakia	703
Europe	Spain	724
Europe	Sweden	752
Europe	Switzerland	756
Europe	The Netherlands	528
Europe	Ukraine	804
Europe	United Kingdom	826
North America	Canada	124
North America	Dominican Republic	214
North America	El Salvador	222
North America	Haiti	332
North America	Honduras	340
North America	Mexico	484
North America	Nicaragua	558
North America	United States of America	840
Oceania	Australia	36
Oceania	New Zealand	554
South America	Argentina	32
South America	Bolivia	68
South America	Brazil	76
South America	Chile	152
South America	Colombia	170
South America	Costa Rica	188
South America	Cuba	192
South America	Ecuador	218
South America	Guatemala	320
South America	Jamaica	388
South America	Panama	591
South America	Paraguay	600
South America	Peru	604
South America	Trinidad and Tobago	780
South America	Uruguay	858
South America	Venezuela	862

Figure S1: Sex-ratios (female:male) of numbers of cancer deaths (all cancer sites excl. NMSC) versus age, based on country-specific raw data in 5-year age categories. Plots are divided by continent and the y-axis is on a log scale. GLOBOCAN 2022.

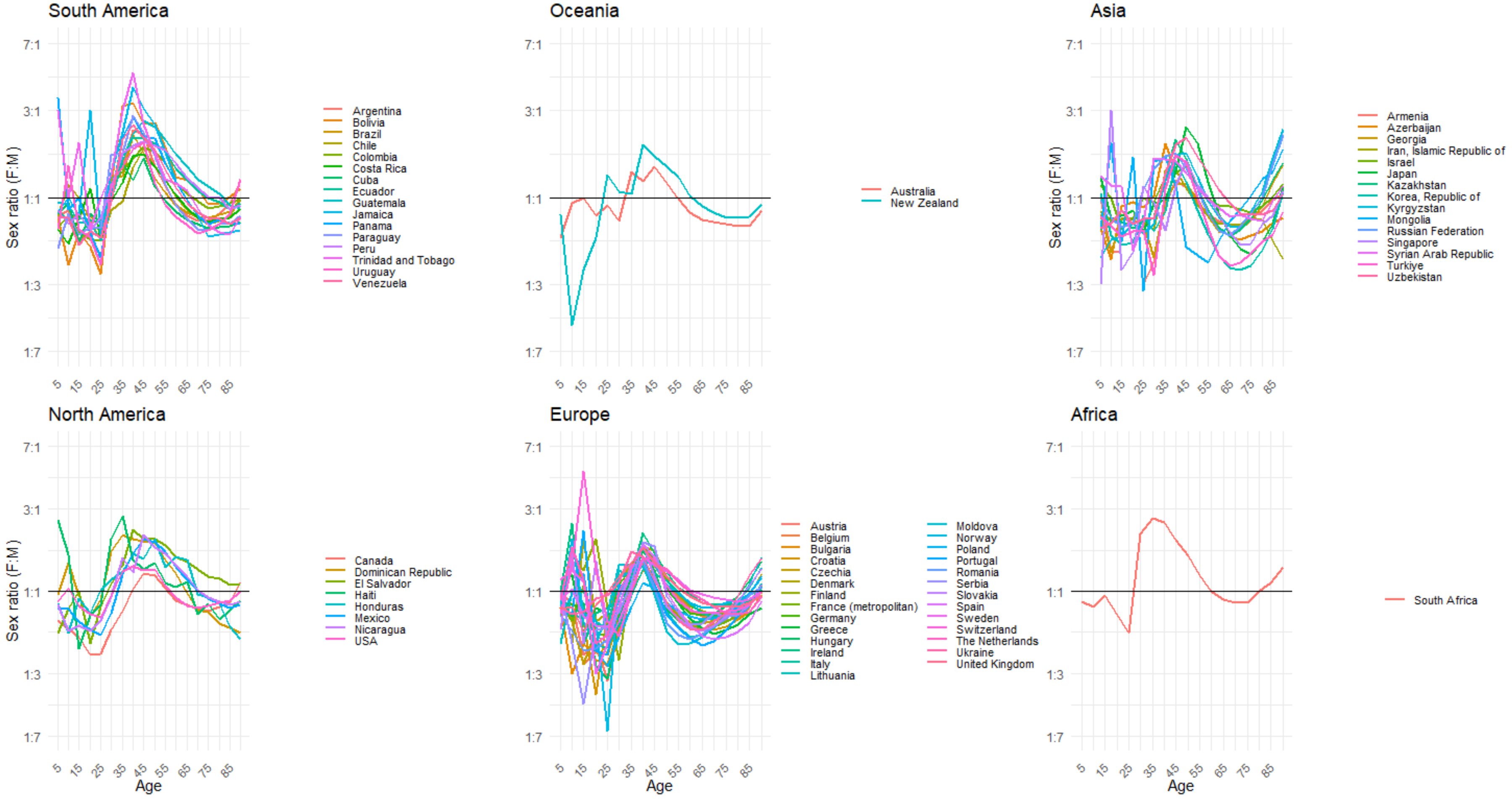

Figure S2 - Proportions of common sexes and sex-specific cancer deaths in global cancer deaths through age groups.

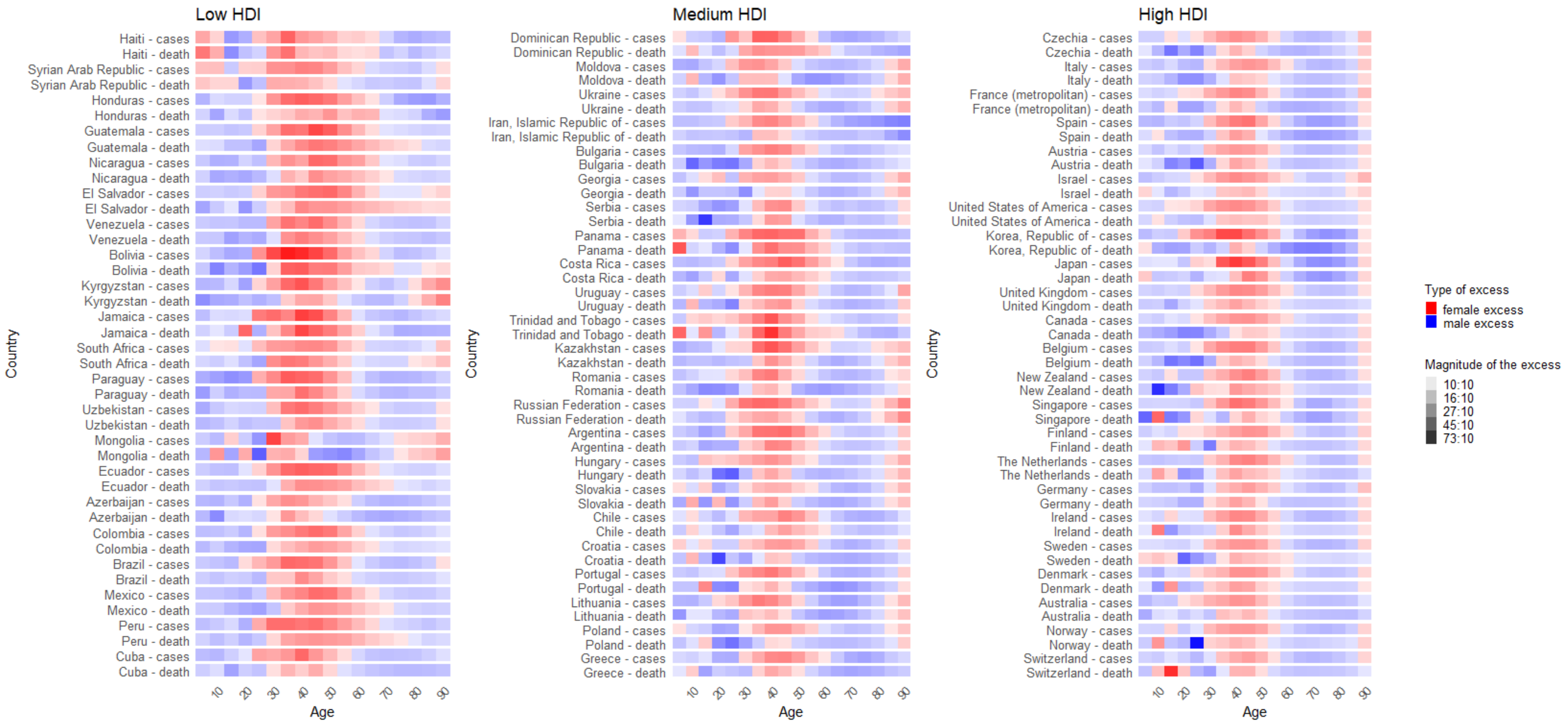

Figure S3: Sex-ratios (female:male) of cancer deaths rates (all cancer sites excl. NMSC) versus age, based on country-specific raw data in 5-year age categories. Plots are divided by continent and the y-axis is on a log scale. GLOBOCAN 2022.

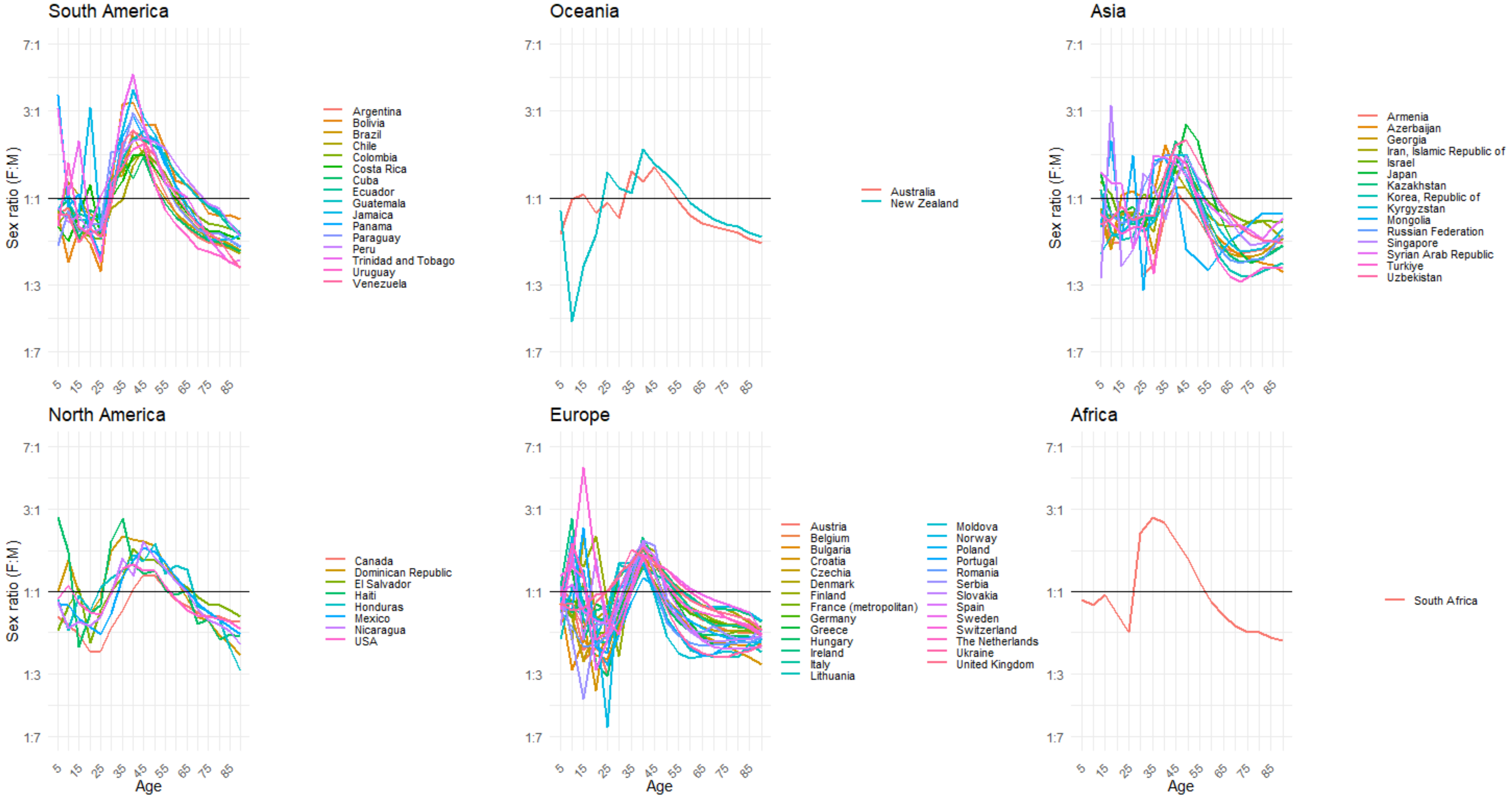

Figure S4: Difference of cases and deaths between females and males, scaled by total number of cases or deaths.

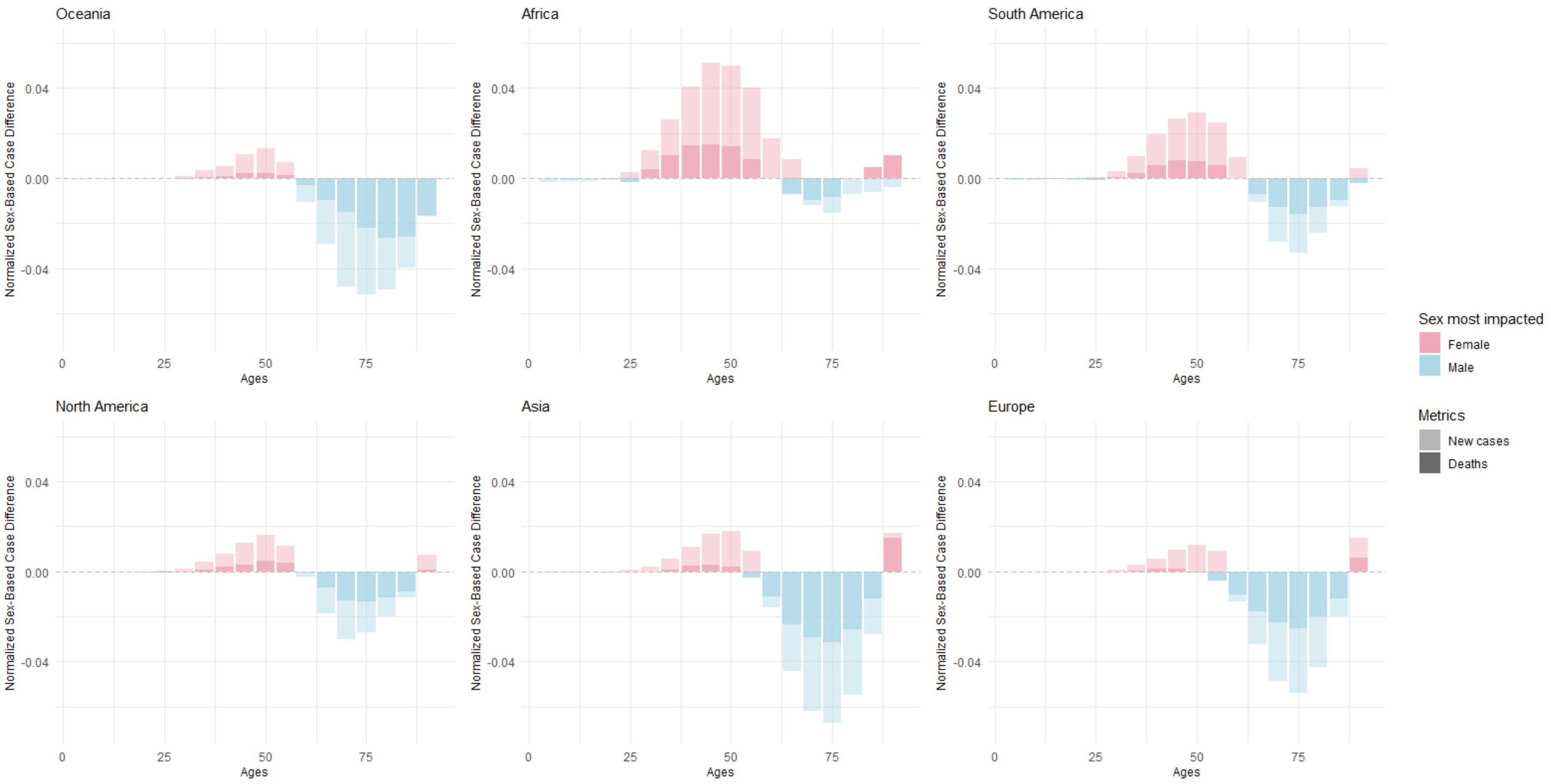

Figure S*5*– Proportions of the age-specific cancer burden by common cancers to both sexes in men and in women: (A) Absolute incidence; and (B) absolute number of deaths.

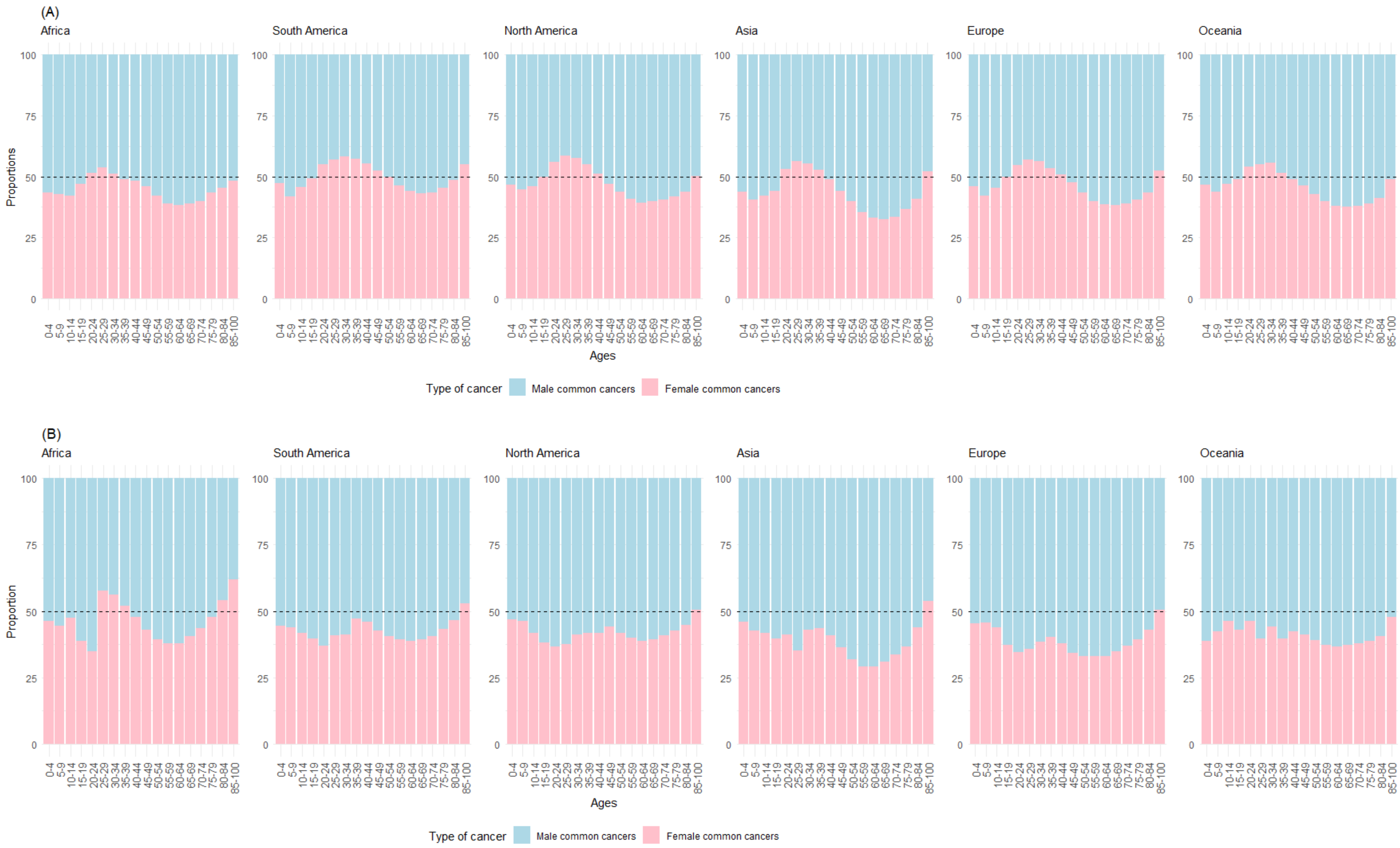
(A)
Africa
South America
North America
Asia
Europe
Oceania
Proportions
100
75
50
25
0
0-4
5-9
10-14
15-19
20-24
25-29
30-34
35-39
40-44
45-49
50-54
55-59
60-64
65-69
70-74
75-79
80-84
85-100
Ages
Type of cancer
Male common cancers
Female common cancers
(B)
Africa
South America
North America
Asia
Europe
Oceania
Proportion
Ages
Type of cancer
Male common cancers
Female common cancers

Figure S6: Sex-ratios (female:male) of absolute numbers of incident cancers (cancers sites common to both sexes) versus age, based on country-specific raw data in 5-year age categories. Plots are divided by continent and the y-axis is on a log scale. Cancer Incidence in Five Continents Vol. X11, 2013-17.

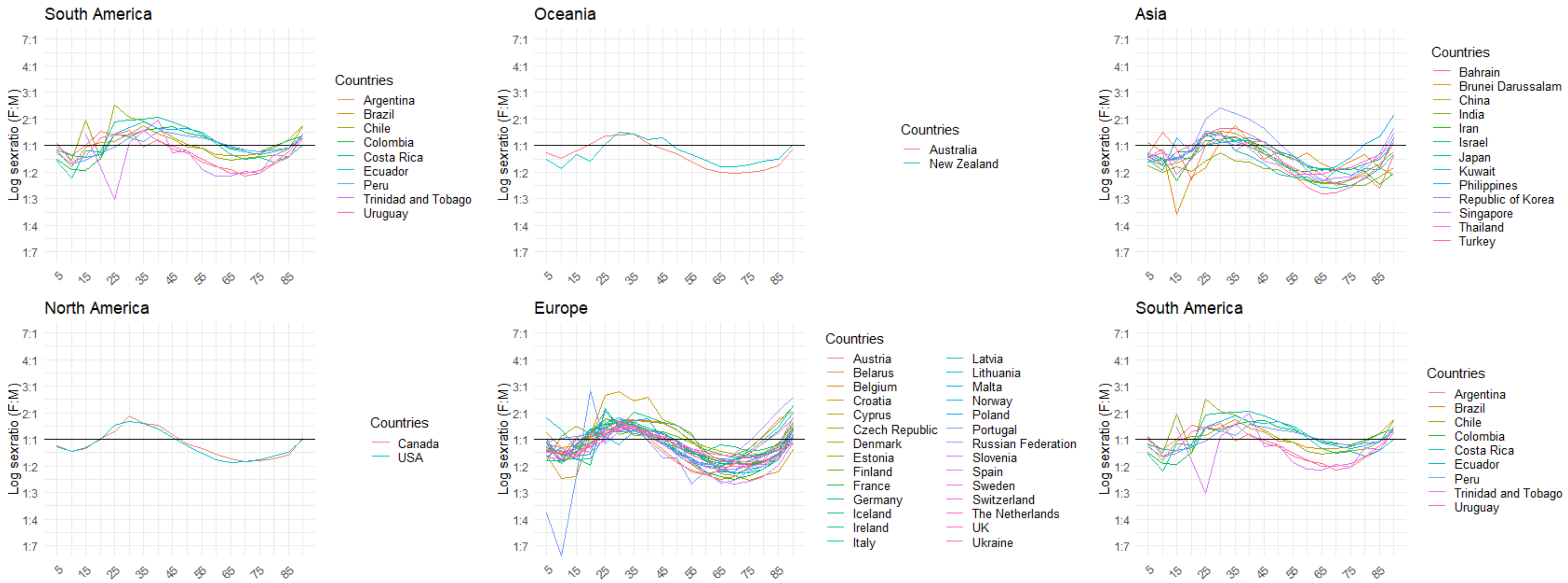

Figure S7: Sex-ratios (female:male) of absolute numbers of deaths (cancers sites common to both sexes) versus age, based on country-specific raw data in 5-year age categories. Plots are divided by continent and the y-axis is on a log scale. GLOBOCAN 2022.

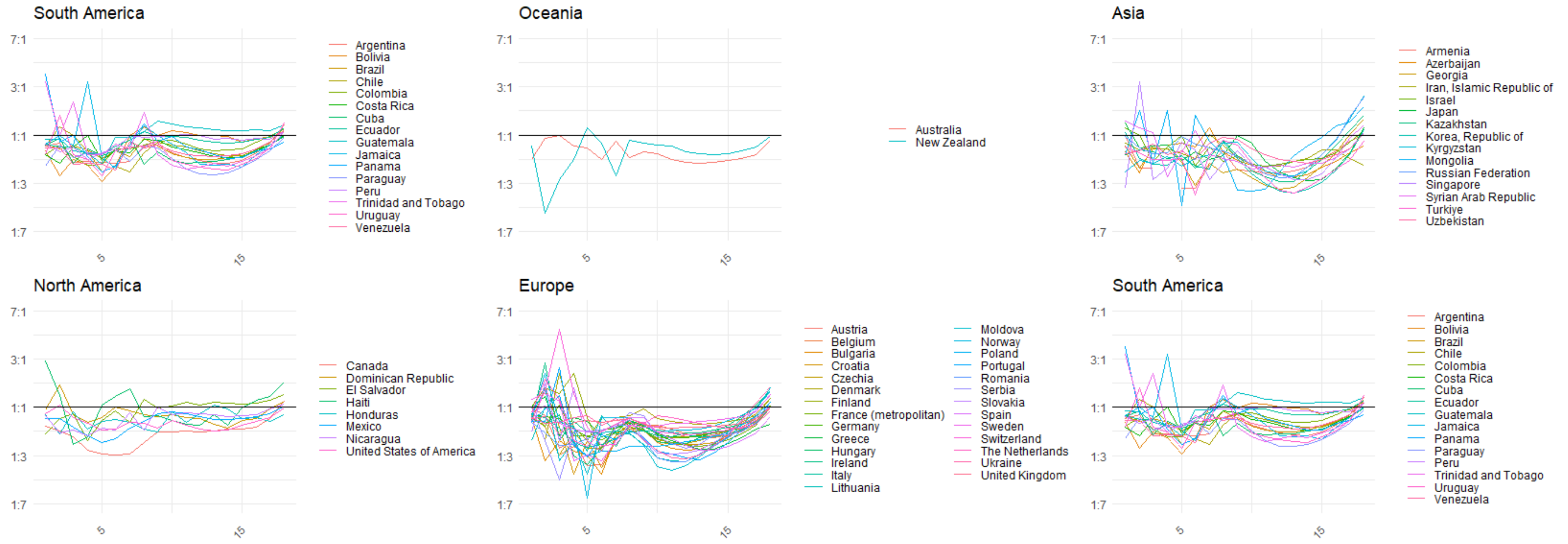

Table S3: Summary table of age-crossovers, maximum sex-ratio and age at the maximum for each country, in incidence.

				Crossover Ages when F:M ratios reverses			Peak F:M and M:F ratios and their timing			
				First	Second	Third	Age		Magnitude	
Continent	*Country*	*Country code*	*Number of AXO*	AXO1	AXO2	AXO3	AM1	AM2	SxR1 (F:M)	SxR2 (F:M)
Africa	Algeria	12	2	15	62	-	44	73	35:10	10:17
Africa	Kenya	404	2	21	66	-	44	84	24:10	10:15
Africa	Mauritius	480	1	21	-	-	44	-	32:10	-
Africa	Morocco	504	2	15	64	-	44	85	40:10	10:16
Africa	Uganda	800	2	17	66	-	54	98	19:10	10:18
Africa	Zimbabwe	716	2	17	70	-	54	85	21:10	10:18
Asia	Bahrain	48	2	24	64	-	49	83	34:10	10:20
Asia	Brunei Darussalam	96	2	20	66	-	39	87	40:10	10:17
Asia	China	156	2	18	54	-	39	71	19:10	10:17
Asia	India	356	2	24	63	-	40	75	17:10	10:15
Asia	Iran, Islamic Republic of	364	2	20	54	-	44	80	20:10	10:22
Asia	Israel	376	3	21	63	83	40	79	21:10	10:12
Asia	Japan	392	3	20	56	89	44	74	26:10	10:20
Asia	Korea, Republic of	410	3	17	56	86	39	69	25:10	10:19
Asia	Kuwait	414	3	20	69	86	44	78	18:10	10:12
Asia	Philippines	608	1	18	-	-	44	-	31:10	-
Asia	Russian Federation	643	3	6	58	73	30	67	27:10	10:11
Asia	Singapore	702	3	22	60	85	44	74	28:10	10:16
Asia	Thailand	764	3	19	61	90	30	70	22:10	10:12
Asia	Turkiye	792	2	22	52	-	43	74	20:10	10:20
Europe	Austria	40	3	24	54	85	44	68	18:10	10:14
Europe	Belarus	112	3	16	52	79	30	65	21:10	10:14
Europe	Belgium	56	3	23	56	87	44	72	23:10	10:15
Europe	Croatia	191	3	28	55	82	44	69	21:10	10:15
Europe	Cyprus	196	2	19	60	-	39	70	29:10	10:19
Europe	Czechia	203	3	20	54	82	44	66	17':10	10:14
Europe	Denmark	208	3	20	58	86	44	74	20:10	10:14
Europe	Estonia	233	3	18	55	79	35	67	21:10	10:15
Europe	Finland	246	3	17	62	85	40	70	20:10	10:15
Europe	France (metropolitan)	250	3	19	55	86	44	67	22:10	10:17
Europe	Germany	276	3	25	57	85	44	74	19:10	10:15
Europe	Iceland	352	3	23	57	88	35	68	28:10	10:14
Europe	Ireland	372	3	15	56	86	36	65	20:10	10:17
Europe	Italy	380	3	23	58	86	40	74	22:10	10:16
Europe	Latvia	428	3	22	56	78	35	68	22:10	10:13
Europe	Lithuania	440	3	17	52	80	35	61	22:10	10:17
Europe	Malta	470	3	30	58	83	43	66	19:10	10:14
Europe	Norway	578	3	25	56	87	44	71	18:10	10:15
Europe	Poland	616	3	21	56	86	35	67	19:10	10:15
Europe	Portugal	620	3	24	50	88	29	64	20:10	10:19
Europe	Slovenia	705	3	27	53	83	44	74	18:10	10:17
Europe	Spain	724	3	25	54	89	44	74	20:10	10:22
Europe	Sweden	752	3	25	56	86	40	65	21:10	10:15
Europe	Switzerland	756	3	28	55	88	40	70	19:10	10:16
Europe	The Netherlands	528	3	27	59	87	40	77	21:10	10:15
Europe	Ukraine	804	3	18	56	80	30	67	22:10	10:11
Europe	United Kingdom	826	3	21	58	88	44	70	20:10	10:14
North America	Canada	124	3	23	59	88	44	72	20:10	10:12
North America	United States of America	840	3	21	57	87	43	66	20:10	10:12
Oceania	Australia	36	2	23	55	-	44	68	17:10	10:15
Oceania	New Zealand	554	3	24	58	88	40	71	22:10	10:15
South America	Argentina	32	3	24	62	83	44	74	23:10	10:13
South America	Brazil	76	3	13	59	81	39	74	25:10	10:13
South America	Chile	152	3	29	57	85	35	74	19:10	10:13
South America	Colombia	170	3	22	66	82	44	70	27:10	10:11
South America	Costa Rica	188	2	20	63	-	44	79	30:10	10:14
South America	Ecuador	218	2	20	66	-	40	75	28:10	10:12
South America	Peru	604	3	24	66	89	40	79	31:10	10:12
South America	Trinidad and Tobago	780	2	25	62	-	39	80	47:10	10:15
South America	Uruguay	858	3	24	57	85	44	70	21:10	10:14

Table S4: Summary table of age-crossovers, maximum sex-ratio and age at the maximum for each country, in mortality.

			Crossover ages when F:M ratios reverses			Peak F:M and M:F ratios and their timing			
			First	Second	Third	Ages		Magnitude	
Continent	*Country*	*Number of AXO*	*AXO1*	*AXO2*	*AXO3*	*AM1*	*AM2*	*SxR1 (F:M)*	*SxR2 (F:M)*
Africa	South Africa	3	25	57	77	30	69	27:10	10:11
Asia	Armenia	3	34	46	86	35	65	13:10	10:14
Asia	Azerbaijan	2	27	46	-	30	69	22:10	10:17
Asia	Georgia	3	33	47	79	44	64	11:10	10:16
Asia	Iran, Islamic Republic of	2	30	45	-	34	85	14:10	10:24
Asia	Israel	3	29	52	82	44	72	15:10	10:12
Asia	Japan	3	33	55	89	40	74	26:10	10:21
Asia	Kazakhstan	3	30	50	78	35	60	23:10	10:16
Asia	Korea, Republic of	3	34	46	89	35	69	17:10	10:25
Asia	Kyrgyzstan	3	31	52	77	44	66	17:10	10:14
Asia	Mongolia	3	26	39	72	34	54	17:10	10:23
Asia	Russian Federation	3	22	49	78	39	64	17:10	10:16
Asia	Singapore	3	36	53	88	40	70	18:10	10:19
Asia	Syrian Arab Republic	3	25	52	85	25	73	16:10	10:13
Asia	Tajikistan	3	30	57	88	46	77	15:10	10:15
Asia	Uzbekistan	3	30	60	86	44	70	22:10	10:12
Europe	Austria	3	32	51	89	35	60	18:10	10:14
Europe	Belgium	3	31	51	88	35	69	16:10	10:14
Europe	Bulgaria	2	32	46	-	35	64	16:10	10:16
Europe	Croatia	2	33	45	-	35	66	13:10	10:17
Europe	Czechia	3	32	46	83	35	61	17:10	10:15
Europe	Denmark	3	26	54	90	35	75	18:10	10:12
Europe	Finland	3	32	52	88	35	69	15:10	10:13
Europe	France (metropolitan)	3	32	45	89	35	60	16:10	10:16
Europe	Germany	3	27	49	88	35	65	19:10	10:13
Europe	Greece	2	31	49	-	35	66	18:10	10:17
Europe	Hungary	3	30	45	79	39	55	18:10	10:15
Europe	Ireland	3	30	55	91	35	74	24:10	10:12
Europe	Italy	3	33	52	88	35	65	13:10	10:14
Europe	Lithuania	3	26	44	81	39	55	14:10	10:18
Europe	Moldova	3	25	42	81	30	51	14:10	10:20
Europe	Norway	3	27	51	85	35	61	15:10	10:12
Europe	Poland	3	36	44	83	39	65	11:10	10:14
Europe	Portugal	3	27	44	88	39	60	17:10	10:21
Europe	Romania	3	29	43	91	39	59	15:10	10:19
Europe	Serbia	3	31	47	88	35	60	20:10	10:16
Europe	Slovakia	3	30	45	81	39	63	20:10	10:17
Europe	Spain	2	30	48	-	35	65	20:10	10:19
Europe	Sweden	2	32	59	-	35	76	16:10	10:11
Europe	Switzerland	2	33	50	-	44	65	15:10	10:13
Europe	The Netherlands	2	28	57	-	35	75	19:10	10:13
Europe	Ukraine	3	24	45	79	30	60	17:10	10:16
Europe	United Kingdom	2	23	54	-	35	75	19:10	10:12
North America	Canada	3	37	54	89	42	74	12:10	10:13
North America	Dominican Republic	2	24	62	-	30	85	21:10	10:18
North America	El Salvador	1	26	-	-	39	-	23:10	-
North America	Haiti	2	24	64	-	34	79	30:10	10:16
North America	Honduras	2	22	67	-	49	85	24:10	10:20
North America	Mexico	2	30	68	-	40	85	20:10	10:12
North America	Nicaragua	2	28	68	-	40	80	23:10	10:12
North America	United States of America	3	29	55	89	35	69	14:10	10:12
Oceania	Australia	2	30	52	-	44	80	16:10	10:14
Oceania	New Zealand	2	21	59	-	35	77	21:10	10:13
South America	Argentina	3	26	56	85	35	70	24:10	10:13
South America	Bolivia	3	27	70	82	39	70	33:10	10:11
South America	Brazil	2	26	55	-	35	73	24:10	10:12
South America	Chile	2	33	58	-	40	70	20:10	10:14
South America	Colombia	2	27	65	-	40	70	19:10	10:11
South America	Costa Rica	2	29	59	-	44	73	17:10	10:13
South America	Cuba	2	27	51	-	44	72	16:10	10:14
South America	Ecuador	2	28	72	-	35	85	21:10	10:12
South America	Guatemala	2	26	79	-	40	85	27:10	10:13
South America	Jamaica	2	26	62	-	35	74	46:10	10:16
South America	Panama	2	28	62	-	35	85	31:10	10:14
South America	Paraguay	2	25	55	-	35	72	30:10	10:15
South America	Peru	2	26	76	-	44	81	20:10	10:16
South America	Trinidad and Tobago	2	23	65	-	39	80	57:10	10:16
South America	Uruguay	3	27	51	88	44	65	20:10	10:16
South America	Venezuela	2	28	60	-	35	81	26:10	10:14
